# Prenatal noise stress impairs HPA axis and cognitive performance in mice

**DOI:** 10.1038/s41598-017-09799-6

**Published:** 2017-09-05

**Authors:** Zahra Jafari, Jogender Mehla, Bryan E. Kolb, Majid H. Mohajerani

**Affiliations:** 1 Department of Neuroscience, Canadian Centre for Behavioural Neuroscience (CCBN), University of Lethbridge, Lethbridge, AB T1K 3M4 Canada; 2 School of Rehabilitation Sciences, Iran University of Medical Science (IUMS), Tehran, Iran

## Abstract

Noise stress is a common environmental pollutant whose adverse effect on offspring performance has been less studied. This study was novel in terms of using “noise” as a prenatal stress compared with physical stress to explore the effect of stress during gestation on HPA axis activation, cognitive performance, and motor coordination, as well as in investigating the effect of behavioral assessments on the corticosterone (CORT) levels. Three groups of C57BL/6 mice with a gestational history of either noise stress (NS), physical stress (PS), or no stress were examined in several behavioral tests. Plasma CORT level was significantly higher before starting the behavioral tests in NS group than the two other groups. It was significantly increased after the behavioral tests in both prenatal stressed groups relative to the controls. Stress caused anxiety-like behavior and reduced learning and memory performance in both stressed groups compared to the controls, as well as decreased motor coordination in the NS group relative to the other groups. The findings suggested that: prenatal NS severely changes the HPA axis; both prenatal stressors, and particularly NS, negatively impair the offspring’s cognitive and motor performance; and, they also cause a strong susceptibility to interpret environmental experiences as stressful conditions.

## Introduction

The early life is one of the most important and sensitive periods during the development of an individual. A large body of evidence supports the conclusion that there are diverse effects of stress during gestation on the offspring’s brain and behavior in both human and non-human animals^1–3^. In humans, the relationship between prenatal stress and increased tendency for developing diverse psychosocial problems has been demonstrated in both childhood and adulthood. For example, the link between prenatal stress and cognitive, behavioral, physical, and emotional problems such as autism and attention-deficit hyperactivity disorder in children, as well as the relationship between prenatal stress and depression and schizophrenia in adulthood, has been well-documented in numerous studies^1, 4, 5^. Studies of laboratory rodents have shown negative neurodevelopmental, behavioral, neuroanatomical, neurochemical, and epigenetic outcomes subsequent to prenatal stress. The behavioral symptoms are broad including anxiety and depressive-like behavior, learning and memory deficits, distractibility and attention disorders, and non-directed locomotor behavior^2, 4, 5^.

Fetal development is regulated by hormones that are transported through the placenta^6^. Stress may also negatively affect the developing immune system leading to the higher incidence of respiratory and other infectious diseases^5, 7, 8^. In addition, maternal stress can influence fetal brain development by constricting the placental arteries and consequently reducing fetal blood flow and the supply of essential nutrients and oxygen^9^. Catecholamines, corticotropin releasing hormone (CRH), and adrenal steroids are stress hormones that penetrate the fetal brain from the maternal circulation. In addition to CRH, which is only shown in primates, other circulating stress hormones including cortisol, corticosterone (CORT), adrenocorticotropic hormone, and aldosterone are increased owing to maternal stress in both the mother and fetuses of rodents and non-human primates^5^. These hormones could then produce alterations in the brain structure and function depending on the hormone type, and amount and time of exposure.

The kind of stressor experienced is an important issue to address in considering the effect of prenatal stress on the offspring’s brain and behavior. Laboratory studies have used a wide range of stressors including crowding, social isolation, food deprivation, cage tilting, exposure to cold, intermittent electric shocks, forced swimming, and restraint alone or restraint with heat and bright light, and noise^2, 4, 6^. Among the diverse types of prenatal stressors, restraint is the most commonly used and noise is the least used stressor in rodent studies^2^. These two stressors differ in how much they are unpredictable and/or uncontrollable and thus likely differ in their induction of the hypothalamic–pituitary–adrenal (HPA) response in animals.

In a study on offspring of pregnant rats exposed to restraint, forced swimming, and crowding, although each stressor significantly increased the plasma CORT level compared with the controls (52 ng/mL), the increases varied by stressors including restraint (522 ng/mL), forced swimming (438 ng/mL), and crowding (247 ng/mL) respectively. The degree of control that an animal perceives it has over the stressful event is one of the important factors that differs among these stressors. Likely, the highest HPA axis response in restraint stress is the result of absolutely no control over the situation for the animal, whereas in both forced swimming or crowding the animal is able to move and therefore has a certain degree of control over the situation^10^. Studies in both humans and laboratory animals have shown that the sense of control over the stressor effects the prefrontal cortex regulation of stress hormones^11^.

Noise is an unwanted unpleasant auditory stimulus whose adverse effects on health have been less studied^12^. The most investigated non-auditory effects of noise on human health are perceived disturbance and annoyance, cognitive impairment (loss of learning and memory), sleep disturbance, and cardiovascular diseases^13, 14^. Noise interferes with daily activities, sleep, or rest, and can induce annoyance and negative responses, such as anger, displeasure, exhaustion, and stress-related symptoms^15^. Stressful noise causes the release of stress hormones, including catecholamines and glucocorticoids^16^. Although a growing body of evidence supports the conclusion that prenatal stressors produce adverse effects on brain development, neuroendocrine function, and behavior in offspring, only a few studies have examined the impact of noise stress on brain and behavior in rodents, particularly in the mouse. The present study was performed to compare the effect of a prenatal noise stress and physical stressors on cognitive and motor performance as well as the HPA axis activity in offspring. We also investigated how the HPA axis activity is modulated by training the mice on a set of behavioral tests.

## Results

No significant differences were observed between the two control groups in any measures (p > 0.05). Therefore, the control groups pooled for results.

### CORT levels

#### The CORT levels comparison before starting and after finishing the behavioral tests

**Figure 1:**
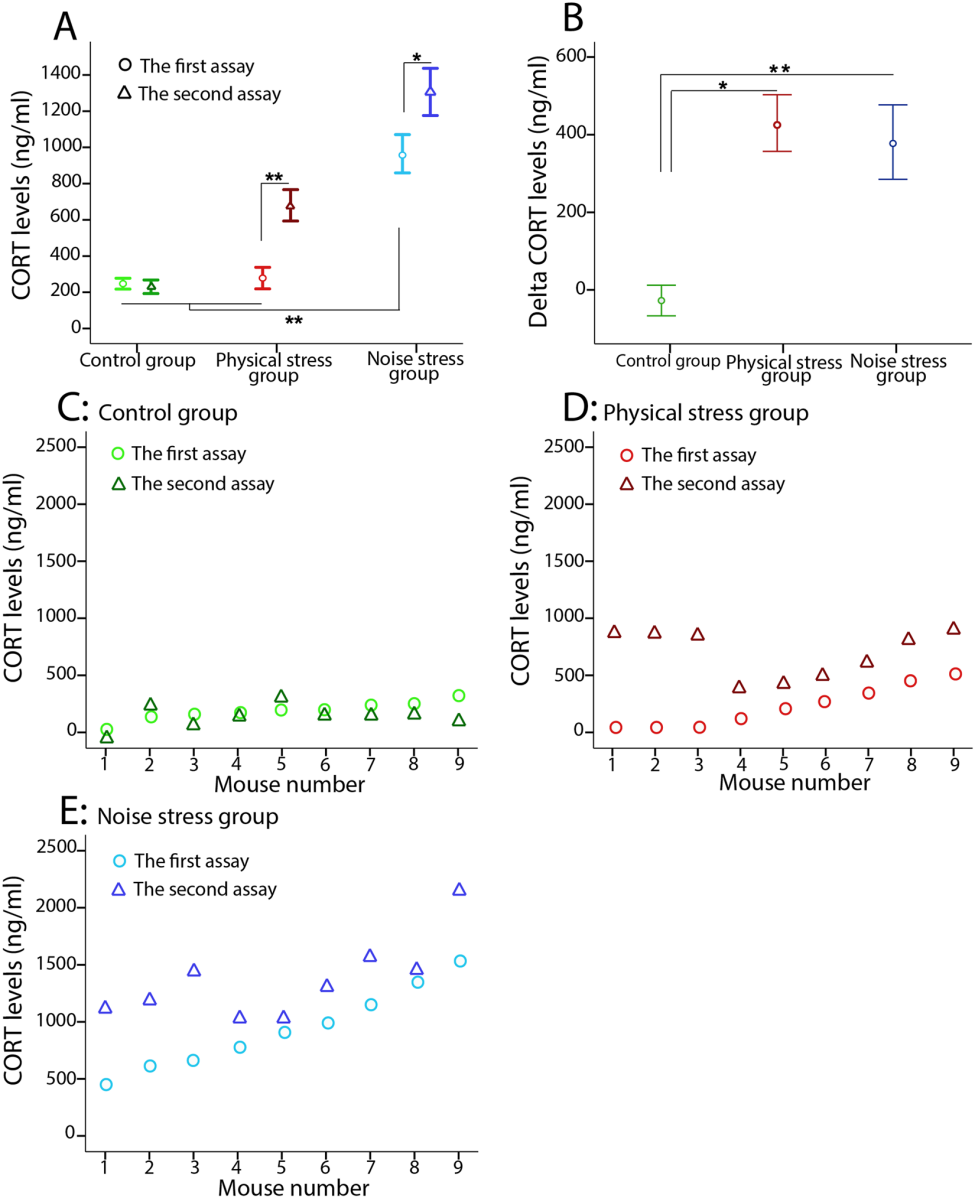
The CORT levels (ng/ml): (**A**) the CORT level in the first assay (○ = before starting behavioral tests) was significantly higher in the NS group compared with the two other groups. A significant increase in CORT levels were observed in the second assay (Δ = after finishing all behavioral tests) than the first assay in both stressed groups relative to the control group. (**B**) A significant difference was obtained in the delta CORT level between the control group and both stressed groups. Results reported as mean ± S.E.M. Asterisks indicate *p < 0.05 or **p < 0.01. (**C**) No significant difference was shown in CORT levels between the first assay and the second assay in the control group. (**D** and **E**) A significant difference was obtained in CORT levels between the first assay and the second assay in both PS and NS groups. N = 9 per group.

Blood collection was conducted twice one day before starting and one day after finishing all behavioral tests. Table 1 compares the two time periods of the CORT assay in each group. No significant difference was found between the two CORT assays in the control group, but in both stressed groups the plasma CORT levels were significantly higher in the second assay compared with the first assay (Fig. 1A). Figure 1B shows the delta CORT levels (the difference between the CORT levels in the first and the second measures) in the three groups. A significant difference was observed between the control group and both stressed groups in the delta CORT level (Table 1).

**Table 1:** (**A**) Comparison of CORT levels within each group to compare levels before starting (the first assay) and after finishing (the second assay) all behavioral tests. (**B**) Comparison of CORT levels among the three groups for the first assay, the second assay, and the delta CORT.

a) In every group	Mean difference (delta CORT)			t	p	η^2^	power
Control group	−28.017			0.505	0.497	0.059	0.096
Physical stress group	414.674			−4.639	**0.004**	0.676	0.945
Noise stress group	396.218			−3.146	**0.021**	0.602	0.761
**b) Among the groups**	*Between groups’ p-values			**Significant main effects			
	**Control and PS**	**Control and NS**	**PS and NS**	**F**	**p**	**η** ^**2**^	**power**
The first assay	0.439	**<0.001**	**<0.001**	23.069	**<0.001**	0.658	1.000
The second assay	**0.021**	**<0.001**	**<0.001**	22.226	**<0.001**	0.649	1.000
Delta CORT	**0.005**	**0.020**	0.563	5.307	**0.012**	0.370	0.787

NS: noise stress, PS: physical stress, η^2^ = effect size, *the “between groups’ p-values” show p-values for the between group comparisons, **the “significant main effects” indicate the statistical results of a significant main effect for every measure.

#### The CORT levels comparison among the three groups

As Table 1 shows, the CORT level was significantly higher in the noise stress (NS) group relative to the other groups in the first assay. After finishing the behavioral tests, the CORT levels significantly increased in both stressed groups compared to the control group. Figures 1C, 1D, and 1E illustrate the CORT levels of every animal twice (pre and post behavior measures) in each group: control group (n = 9), physical stress (PS) group (n = 9), and in the NS group (n = 9), respectively. In the Fig. 1D and E, animals were ordered from the lowest to the highest CORT levels measured before the behavioral tests.

### Behavioral tests

#### Novel object recognition (NOR) test

**Figure 2:**
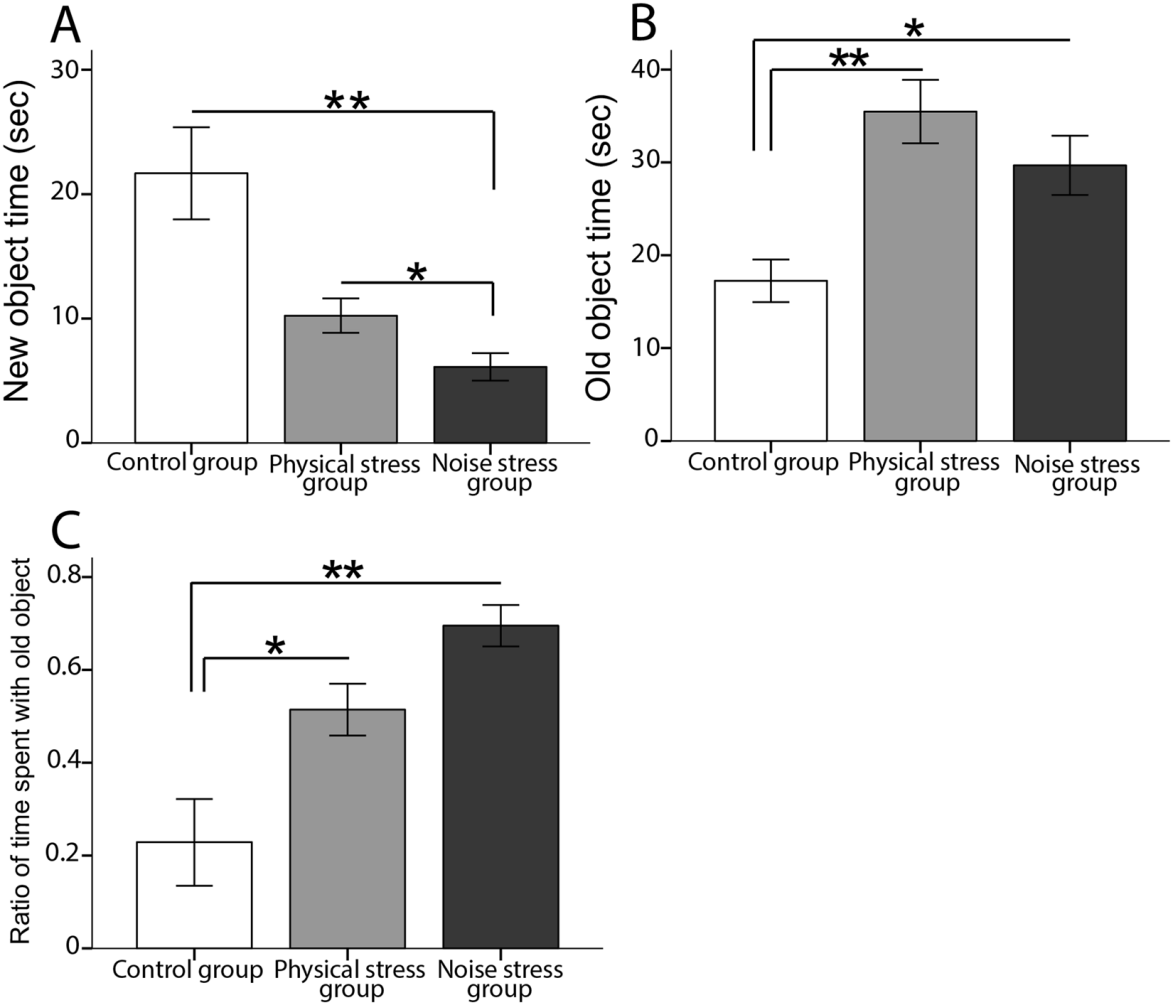
The NOR test: (**A**) The NS group significantly spent shorter time (sec) with the new object compared with the two other groups. (**B**) Both stressed groups significantly spent longer time (sec) with the old object relative to the control group. (**C**) The ratio of time spent with old versus new object was significantly higher in both stressed group than the control group. N = 10 per group. Results reported as mean ± S.E.M. Asterisks indicate *p < 0.05 or **p < 0.01.

The NS group revealed a significantly shorter time spent with the new object compared with the two other groups (Table 2). Both stressed groups showed a significantly longer time spent with the old object than the control group. The ratio of time spent with old compared to new object was significantly higher in both stressed groups than the control group (Fig. 2). The difference among the groups was not significant in any measures of the locomotion behavior including movement time (F_2,27_ = 0.577, p = 0.571, η^2^ = 0.060, power = 0.131), movement number (F_2,27_ = 0.643, p = 0.537, η^2^ = 0.067, power = 0.141), total distance (F_2,27_ = 1.012, p = 0.385, η^2^ = 0.094, power = 0.203), or rest time (F_2,27_ = 0.913, p = 0.401, η^2^ = 0.101, power = 0.198).

**Table 2:** Comparison among the three groups in different measures for all behavioral tests.

NOR test	*Between groups’ p-values			**Significant main effects			
	Control and PS	Control and NS	PS and NS	F	p	η^2^	power
New object time (sec)	0.657	**0.009**	**0.010**	6.452	**0.005**	0.315	0.871
Old object time (sec)	**0.001**	**0.038**	0.151	5.233	**0.012**	0.275	0.789
Ratio of old object time (%)	**0.022**	**0.001**	0.098	6.903	**0.004**	0.330	0.893
**EPM test**							
Open arm time (sec)	**<0.001**	**<0.001**	0.129	14.094	**<0.001**	0.493	0.997
Closed arm time (sec)	**0.025**	**0.032**	0.896	3.225	**0.048**	0.202	0.597
Number of entries to open arm	**0.001**	**<0.001**	0.102	13.686	**<0.001**	0.486	0.996
Number of entries to closed arm	0.139	**<0.001**	**0.022**	3.772	**0.035**	0.206	0.642
**BBT**							
Number of foot slips	0.100	**0.007**	0.148	4.273	**0.021**	0.257	0.732
Number of turns	0.378	**0.044**	0.143	2.780	0.111	0.153	0.456
**MWT**							
Swim time (sec)	**0.004**	**0.014**	0.784	5.481	**0.004**	0.011	0.850
Swim speed (m/s)	**<0.001**	**0.001**	0.390	10.416	**<0.001**	0.022	0.988

BBT: balance beam test; EPM: elevated plus maze; MWT: Morris water task, NOR: novel object recognition, NS: noise stress, PS: physical stress, η^2^ = Effect size, *the “between groups’ p-values” show p-values for the between group comparisons, **the “significant main effects” indicate the statistical results of a significant main effect for every measure.

#### Elevated plus maze (EPM) test

**Figure 3:**
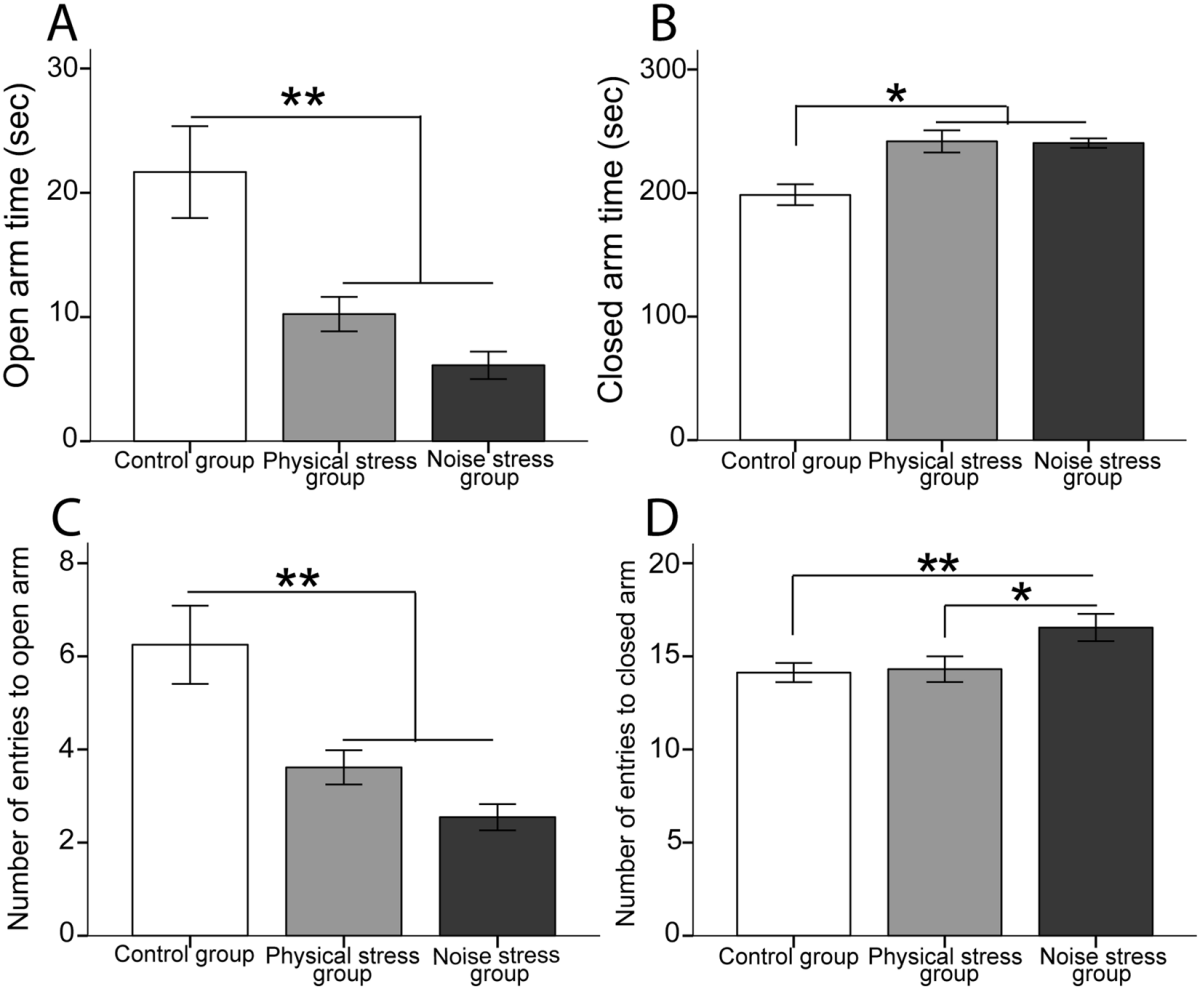
The EPM test: Both stressed groups showed: (**A**) a shorter time in open arms (sec); (**B**) a longer time in closed arms (sec); and, (**C**) a lower number of entries to open arms compared with the control group. (**D**) The NS group revealed higher number of entries to closed arm than the two other groups. N = 10 per group. Results reported as mean ± S.E.M. Asterisks indicate *p < 0.05 or **p < 0.01.

As Table 2 indicates, compared to the control group both stressed groups showed significantly less open arm time (time spent in the open arm; sec), greater closed arm time (time spent in the closed arm; sec), and a lower number of entries to open arms. The NS group had a significantly higher number of entries into closed arms compared with the other groups. Figure 3 compares the means of variables in all groups.

#### Balance beam test (BBT)

**Figure 4:**
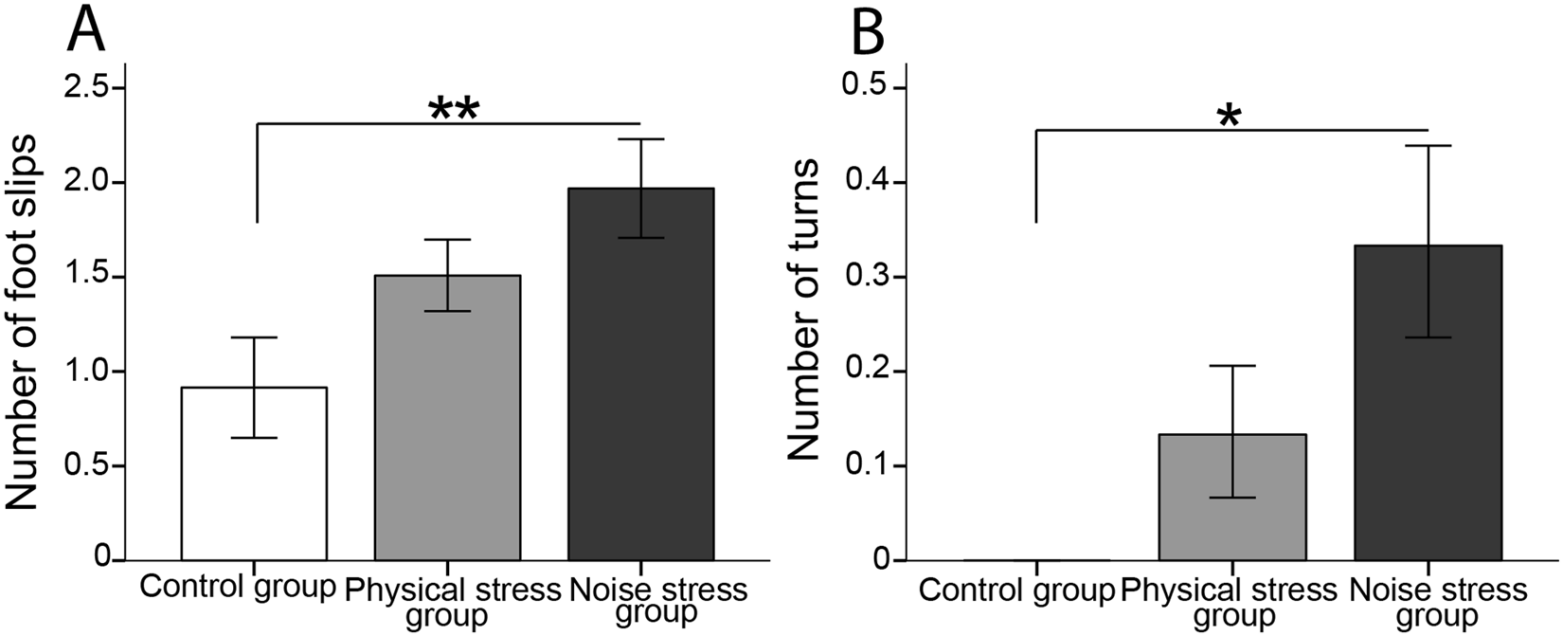
The BBT: The noise stress group revealed significantly (**A**) higher number of foot slips, and (**B**) higher number of turns compared with the two other groups. N = 10 per group. Results reported as mean ± S.E.M. Asterisk indicates *p < 0.05.

The latency to pass across the beam was higher in both stressed groups (PS group mean: 10.39 ± 3.90 sec, NS group: 9.82 ± 2.14 sec) relative to the control group (8.05 ± 2.50), but the difference was not significant (F_2,27_ = 1.612, p = 0.278, η^2^ = 0.091, power = 0.321). The NS group showed a significantly higher number of foot slips, and higher number of turns compared with the other groups (Table 2). Figure 4 shows mean scores of measured variables for the three groups.

### Morris water task (MWT)

#### Swim time

**Figure 5:**
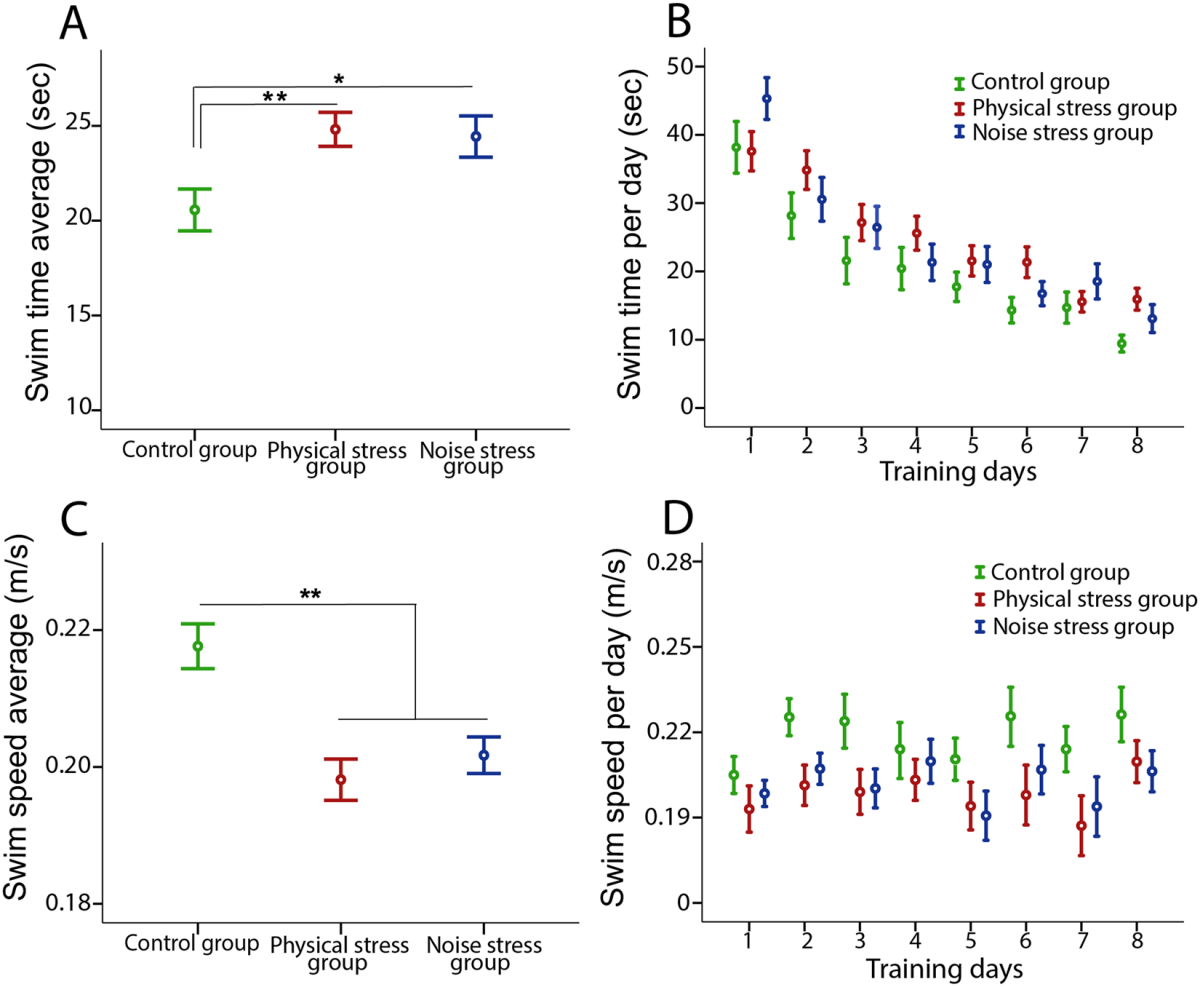
The MWT: (**A**) The swim time average (sec) and (**B**) the swim time during 8-days training in the three groups. A significantly longer swim time was observed in both stressed groups relative to the control group. (**C**) The swim speed average (m/s) and (**D**) the swim speed during 8-days training in the three groups. A significantly lower swim speed was obtained in both stressed groups versus the control group. N = 10 per group. Results reported as mean ± S.E.M. Asterisks indicate **p < 0.01.

Both stressed groups showed significantly longer time to reach the platform relative to the control group. Figure 5A and B illustrates the learning process of the three groups during 8-day course of training in terms of latency to reach platform or swim time (sec). Table 2 compares the three groups in average of swim time during days of training on the MWT. Also in intra-group analysis, a significant difference was observed in swim time across days of training (Control group: F_7,273_ = 14.004, p < 0.001, partial η^2^ = 0.342, power = 1.0; PS group: F_7,273_ = 11.081, p < 0.001, partial η^2^ = 0.633, power = 1.0; NS group: F_7,273_ = 13.469, p = < 0.001, partial η^2^ = 0.771, power = 1.0) in each group.

#### Swim speed

Both stressed groups showed significantly slower swim speed to reach the platform compared with the control group. Figure 5C and D indicates the swim speed (m/s) of the three groups during days of training. Table 2 compares the three groups in average of swim speed (m/s) during course of training of the MWT. In an intra-group analysis, no significant difference was shown in swim speed (m/s) during eight days of training in the three groups (control group: F_7,273_ = 1.694, p = 0.071, partial η^2^ = 0.411, power = 0.765; PS group: F_7,273_ = 0.871, p = 0.537, partial η^2^ = 0.119, power = 0.331; NS group: F_7,273_ = 1.732, p = 0.142, partial η^2^ = 0.302, power = 0.591).

#### Swim distance

Although both stressed groups travelled a longer path (m) to reach the platform compared with the control group, this difference was not significant (F_2,27_ = 1.943, p = 0.143, partial η^2^ = 0.004, power = 0.405).

#### Probe test

Both stressed groups spent shorter time in the target quadrant than the control group, but this difference was not significant (F_2,27_ = 0.657, p = 0.473, partial η^2^ = 0.037, power = 0.136).

## Discussion

The four main findings were: 1) the NS group had a higher baseline CORT level than the other groups; 2) behavioral testing increased the CORT levels in both stress groups but not in the controls; 3) both stress groups performed significantly differently from the control group on the elevated plus maze, novel recognition memory test, and the MWT; and, 4) the performance of the NS group in the motor task was impaired relative to the other groups. We consider these findings in turn.

Corticosterone is a major component of the stress system that along with the sympathetic nervous system plays a prominent role to mobilize energy stores during stressful conditions^4, 17^. In the current study although both stressors increased CORT levels, the NS procedure had almost a fourfold greater effect on offspring’s HPA axis response relative to the PS procedure. Interestingly, in the second measure, where we hypothesized that familiarization with the surrounding environment through the behavioral assessments could act as a positive factor leading to a decrease in the CORT levels, CORT levels were remarkably increased compared to the first measure in both prenatal stress groups. This finding suggests that encountering novel conditions during behavioral assessments was stressful for both prenatal stress groups. High levels of CORT concentrations before starting behavioral assessments in the NS animals could be the result of high levels of maternal CORT levels reaching the fetal brain through the placenta. Numerous studies suggest that prenatal stress is associated with higher baseline CORT release^18–20^. Furthermore, greater stress reactivity^19, 21, 22^ and slower recovery from the stressors^18, 23^ have been indicated in prenatally stressed rodents. The lipophilic structure of CORT naturally allows it to readily pass biological barriers such as the placenta. Although CORT is essential for fetal growth and the induction of certain enzymes to prepare the fetus for extrauterine life, fetal physiological CORT levels are much lower than maternal levels. The fetoplacental 11β-hydroxy steroid dehydrogenase type 2 (11β-HSD2) is an enzyme that catalyses the rapid metabolism of active CORT (cortisol) to physiologically inactive 11-keto forms (cortisone, 11-dehydro corticosterone) and makes a barrier to transport maternal CORTs. This barrier is not fully developed to protect the fetus against extra levels of CORT during stress exposure. Therefore, high proportions of maternal CORT reach to the fetal brain and elevate CORT levels^24^.

CORT is a systemic intercellular signal whose level dynamically increases with environmental and psychological stressors, and is associated with weight loss, adrenal hypertrophy, thymus involution, and reduced corticosteroid binding globulin (CBG), and each of these changes are other hallmark signs of general stress^25^. Given the extreme levels of CORT in the NS group prior to doing the behavioral tests in our study, further investigations on HPA axis-related parameters (i.e., thymus and adrenal weight, ACTH and CBG levels) would be helpful in providing a more comprehensive view regarding the harmful effects of prenatal stress on general health. Hypothalamic structural rearrangement owing to prenatal stress is also gender specific and differs between males and females^26^, that this aspect was not investigated in the present study.

The open field tests, such as EPM, have been used to detect potential anxiety-like behavior resulting from prenatal stress in rodents^2^ and the EPM is likely the most employed animal model of anxiety in past studies^27^. In our study, both prenatal stress groups revealed significantly less time in open arms, longer time in closed arms, and a lower number of entries in open arms than the control group. The NS group also revealed a higher number of entries into closed arms compared the other groups. The number of entries into open arms and time spent in open arms has been suggested as a primary anxiety index^28^. The results also suggest that the noise stress can be stressful enough to produce anxiety-like behavior in offspring similar to that seen after physical stressors. The findings are a replication of numerous publications in offspring of pregnant rodents with the EPM^16, 29–35^.

There was also an effect of prenatal stressors in the NOR test. The tendency to explore a new object more than an old familiar one has been taken as a sensitive test of stimulus recognition memory in rodents^36, 37^. In our study, the NS group spent significantly less time with the new object than the other groups. Both prenatal stress groups also spent significantly longer time with the old object, and their ratio of time spent with old object was significantly higher relative to the control animals. Previous studies suggest maternal stress during a critical period of fetal brain development could highly increase the likelihood of anxiety and depressive disorders. Factors such as the type, timing, and intensity of the maternal stress, as well as gender, age, and test condition also can affect the results^4, 5^. Furthermore, no significant difference was observed among the three groups in any measure of the locomotion behavior, i.e., movement time, movement number, total distance, and rest time. This finding indicates that the increase in the time spent in the closed arms in the EPM is not a result of a decrease in total locomotor activity^27, 29^.

Whereas all groups indicated a significant improvement in learning behavior during eight days-training of the MWT, both prenatal stress groups showed markedly reduced performance in spatial learning compared with the controls. They showed a significantly longer latency and lower speed to reach the platform than the control group, whereas no significant difference was revealed in memory retention. The results thus show an adverse impact of both stress paradigms in spatial learning and allocentric navigation compared with the control mice. Similarly in recent studies, reduced spatial learning, but not memory retention, has been suggested in pre-pubertal and adult male C57/BL mice owing to the prenatal stress^38, 39^. In this regard, many studies have shown retardation and/or alternation of neuronal development in the fetal brain, as well as smaller hippocampal size and reduced number of hippocampal granule cells resulting from high levels of maternal CORT^40–45^. As almost 85% of hippocampal granule cells are created postnatally in rodents, the negative effects of maternal high plasma CORT levels appear to extend long after birth. Several studies also suggested diverse rates of reduction in dendritic arborization and synaptic loss in the CA1 and CA3 areas due to prenatal stress as measured on different postnatal days^41, 46, 47^. Allocentric navigation involves the hippocampus, entorhinal cortex, and surrounding structures^48, 49^, and the interaction of these structures with the prefrontal, anterior cingulate, parietal, and retrosplenial cortices are required for memory consolidation and retrieval^50^. Our results are in agreement with past studies^51–53^ that suggested that prenatal stress negatively affects the neural networks involved in allocentric navigation. It has also been shown to reduce adult hippocampal neurogenesis^54, 55^.

The swim speed of the prenatal stressed groups was also significantly slower than the control group. Previous studies have indicated that regardless of possible confounding factors such as appetite or differences in body weight, rodents typically swim at similar speeds in the MWT, and changes in swim speed owing to a treatment are typically small. Therefore, a difference in swim speed might not account for a difference in learning the platform location^49^. A reduced swim speed could also indicate an increased passive swimming behavior, which can be taken as evidence of increased depressive-like behavior. Using tests sensitive to depressive-like behavior such as the forced swim test (FST) might support such an assumption. The FST, which is one of the most commonly used animal models for assessing depressant-like behavior, has two major behaviors in rodents including active (swimming and struggling) and passive (immobility) behaviors, and the latter one is typically described as depressive-like behavior^56^. In our study, however, a significant slower swim speed, might be related to the impact of the stress procedures on offspring mental state, attentional skills, or mood^56–59^. This needs to be explored further in the future.

Besides glucocorticoids, there are other neuronal regulators that have an important role in the development and survival of neurons in the central nervous system. Brain-derived neurotrophic factor (BDNF) is a growth factor is expressed in the hippocampus^60^, and has a critical role for learning and memory formation^61^. Recent studies in rodents suggest that prenatal stress reduces the levels of BDNF mRNA, presumably under modulation of epigenetic mechanisms^29, 62, 63^. Like BDNF, noradrenaline contributes on learning and memory through activating B-adrenergic receptors and regulating hippocampal and neocortical functions^64, 65^. Noradrenaline and glucocorticoids each has an ‘inverted U-shaped influence on learning and memory, and even a slight change in these neural regulators impairs prefrontal cortex (PFC) and hippocampal functions^11^.

While numerous human and animal studies have demonstrated that prenatal stress affects behavioral, mental, and cognitive development in offspring, few studies have explored the outcomes of prenatal stress on motor development. Recent human studies suggest that prenatal stress or anxiety during gestation negatively modifies infant neuromotor development^66, 67^. A longitudinal study of motor development from the Western Australian Pregnancy (Raine) Study cohort (n = 2,900), which used the McCarron Assessment of Neuromuscular Development, showed a negative relation between the number of stressful events, especially in late pregnancy, and the mean neuromuscular development index measured at 10, 14, and 17 years^67^. In rodents, the BBT has been used as a sensitive test of early detecting balance coordination deficits^68^. The NS group in our study showed a significantly higher number of foot slips and a higher number of turns compared with the control group in the BBT. In a study on pregnant rats exposed to an acute or a repeated stress (presence of a cat) either at the gestational day (GD) 10 or 14, the development of the vibrissae placing response, the righting reflex, and the negative geotaxis behavior was delayed, particularly with a repeated stressor, in the offspring of dams stressed at the GD 10 when offspring were assessed through the first two weeks of life. The delay in motor development was interpreted as an alteration in maturation of nervous structures due to prenatal stress^69^. Another study revealed that hindlimb unloading, a ground-based model widely used to plan the absence of weight support on hindlimbs, severely impaired motor activity and skilled walking in rats. Prenatal stress (restraint for 45 min three times daily from GD11 until delivery) exacerbated the effects of unloading, owing to immaturity of sensorimotor systems in response to environmental challenges during adulthood^70^. The authors concluded that a steady prenatal environment is necessary for normal development of the sensorimotor systems. In contrast, in a study on Swiss mice, Pallares *et al*.^71^ found that prenatal stress (restraint under bright light for 45 min, three times per day from the GD15 until birth), led to improvement in motor performance of the offspring in the BBT and this effect was sex and age dependent. The authors proposed that the prenatal stress had some type of adaptive or protective effect on motor functions. These two inconsistent studies used different durations of prenatal stress and used different species suggesting that further studies are needed to clarify effects of prenatal stress on motor development. We believe functional assessment of brain dynamics using optogenetic and neuroimaging tools^72–75^ combined with animal behaviour is ideally suited to monitor in real time the process by which prenatal stress changes the structure and function of different brain circuits.

## Conclusion

This study compared the effects of prenatal physical stressors and “noise” stress on cognitive and motor performance as well as CORT levels. There was a higher level of CORT before starting behavioral assessments in the NS group compared to the other groups. Both stressed groups had increased CORT effects after behavioral testing. The results of behavioral assessments revealed signs of anxiety-like behavior and reduced learning and memory performance in the prenatal stress groups relative to the controls. Decreased motor coordination was only observed in the NS group. Overall, the results imply loud noise exposure during gestation negatively affects the HPA axis as well as cognitive and motor performance as large as or stronger than the physical stressors in the adult offspring.

## Methods

### Animals

All experiments were carried out in accordance with the Canadian Council of Animal Care and approved by the University of Lethbridge Animal Care Committee. All animals were given access to food and water ad libitum and were maintained on a 12:12-h light:dark cycle in a temperature-controlled breeding room (21 °C) with less than 66 ± 2 dBC room noise level. Thirty female C57BL/6 mice between 8 to 11 weeks of age were individually mated with thirty male C57BL/6 mice in standard shoe-box cages at 4:00 pm. For recording of gestational length, the female mice were assessed three hours later at 7:00 pm and the next morning for breeding signs such as sperm plug and red/swollen vaginal opening ^16^. If a plug or sperm was present, the female was considered possibly pregnant and removed from the male. Once a female was left with a male overnight, she was not paired with a male again until the lack of pregnancy was confirmed. The weight gain of the female mice was followed every day to confirm pregnancy. On the gestational day (GD) 11, a weight gain of at least 3.5 g usually signifies that conception has occurred. This method allows a determination of the length of gestation with a 0.5-day precision^6^. When the pups were born, the dams were kept individually with the litters. At the age of 21–23 days, the pups were weaned from their mothers, and only one male pup was randomly selected from each litter (n = 10 in each group).

### Experimental design

#### Stress Procedure

Pregnant mice were randomly assigned to three groups consisting of two stress groups and one control group. We exposed animals to stress on GDs 12–16 because the corticogenesis process occurs from embryonic day 10 to 17 in mice, and the layer II/III, IV, and V mainly develop during GDs 12–16^76, 77^. This timeframe also corresponds to the second trimester in human pregnancy when substantial neural development occurs^78^.

### Physical stress group

Two stressors, restraint and elevated platform (EP), were applied daily from GDs 12 through 16^79^. For restraint, mice (n = 10) were maintained in a transparent Plexiglas container (5 cm inner diameter), 20 minutes per day at 10:00 am. The container maintained the mice in a standing position without compression of the body^80, 81^. For the EP stressor, each mouse was placed on an elevated platform (1 m height, 21 × 21 cm), 30 minutes twice a day at 9:00 am and 3:00 pm^47, 82^.

### Noise stress group

On GDs 12, 14, and 16, a female pregnant mouse was transferred into a standard cage and moved to a sound chamber. A speaker, which emitted an intermittent 3000 Hz frequency sound of 90 dB^7, 83, 84^ for 1 sec duration and 15 sec inter-stimulus interval (ISI)^85^, was placed in the cage. The sound pressure level was measured daily inside the cage without an animal (Tektronix RM3000, Digital Phosphor Oscilloscope). The mice (n = 10) were exposed to the NS for 24 hrs starting at 8:00 am^6, 79^. A similar protocol was previously used in rats, including a low-frequency sound of 300 Hz for 1 sec in the intervals of 15 sec during 24 hrs.^85^. Here we used a 3000 Hz frequency tone, since (a) is audible by mice^86, 87^ and (b) is relatively similar to environmental and traffic noises which are largely made up of low to mid frequency tones^88, 89^. The intensity of NS exposure used in previous studies was also between 95 to 130 dB^7, 16, 83, 84^. We applied an intermittent stimulus intensity (90 dB) to prevent noise-induced hearing loss. In addition, 24 hrs rest after every stress exposure provided enough time for to recover from possible temporary threshold shifts^90^.

### Control group

There were two sets of control animals: one served as a control for NS dams and another was a control for PS dams. In NS control group, pregnant mice (n = 5) on GDs 12, 14, and 16 were individually transferred into a standard cage and moved to a sound chamber. A silent speaker was placed in the cage. The mice were left undisturbed for 24 hrs starting at 8:00 am. In PS control group, pregnant mice (n = 5) were removed daily from the home cage for 20 or 30 minutes (depending on the type of stressor) during GDs 12–16, transferred to the same testing room for the PS, left undisturbed, and then returned to their home cages. In the control group, no stress was given.

### Plasma CORT assay

The blood sampling procedure was performed at 7:30 to 8:30 am^91^ one day before starting behavioral tests and a day after finishing all behavioral tests. Blood was taken from the submandibular vein^92^ for all animals by one of the authors (ZJ) in order to control for any possible effects of experience and/or inter-tester difference on the results. The submandibular bleeding of mice is a single-use lancet method to quickly draw blood without the use of the anesthesia^92^. No obvious difference in the response of the different animals to the procedure was observed. Approximately 0.1 ml of submandibular blood was collected in heparin-coated tubes. These tubes were centrifuged at 6000 rpm at 4 °C for 15 min to collect the plasma^93^. Collected plasma samples then were stored at −80 °C until further analysis. A commercially available ELISA kit was used to quantify the levels of CORT in the plasma. The assay was carried out as per the manufacturer’s instructions^79^. The optical density of CORT was read at 450 nm wavelength using a microplate reader (Synergy HT BioTek®). The concentration of CORT in samples was calculated using KC4 Bio-Tek® Microplate Data Collection and Analysis software. To reduce intra-plate variability, the coefficient of variation (CV) for all samples was determined using the same standards and controls across all plates, and only samples with a CV less than 10 percent were included in the analysis. The CORT concentration in plasma samples was expressed in ng/ml^16, 91, 94–97^.

### Behavioral assessments

**Figure 6:**
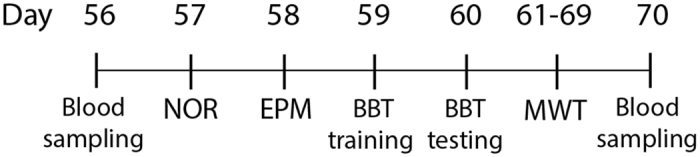
Time course of blood samplings and behavioral tests: The first blood sampling was performed when pups reached 8 weeks old (56 days). After doing a set of behavioral tests including NOR (novel object recognition), EPM (elevated plus maze), BBT (balance beam test), MWT (Morris water task), and probe test, the second blood sampling was conducted a day after the last behavioral test (70 days).

Several behavioral tests were done when pups reached 8 weeks old to measure the effect of prenatal stress on cognitive and motor performance of the offspring. Tests of NOR, EPM, BBT, and MWT, including a probe trial, were conducted respectively in separate days, only one test per day, with an alternating order of animals, by the same examiner in the mornings at 9–11am (Fig. 6).

### Novel object recognition (NOR) test

Each mouse was placed in an open-field arena (47 cm width × 50 cm length × 30 cm height) made of white Plexiglas. In the first trial, the mouse was placed in the arena with two identical objects and was allowed to explore the field for 5 min. The animal was removed and placed in a transport box for 3 min, and one of the objects was randomly replaced with a new object. The mouse was then returned to the arena, and the animal’s exploration was filmed (30 frames/second) for 3 minutes. The time spent with each object was only calculated during the second session. If the nose of the mouse was within 1 cm of the object, it was considered to be in contact with an object^6, 82, 98^. The ratio of time spent with the old compared to the new object was calculated by subtracting times spent with old from the new object divided by the total time spent for exploration^99^. In addition, behavioural measures including movement time (the time in seconds spent moving in the arena), movement number (the number of movements after the animal remained immobile for more than one second), total distance (the total length of paths traveled by animals, in centimeters), and rest time (the time in seconds spent immobile) during the second session were calculated to determine the locomotor behavior of the animals.

### Elevated plus maze (EPM) test

The EPM has been constructed from black Plexiglas®, with a base measuring 48 cm high. The two open arms were 5 cm wide and 27 cm long. The two closed arms were 5 cm wide, 27 cm long, and had walls measuring 21 cm high. The maze was housed in an empty room and a dim light was used during filming. The camera for filming was placed at the end of an open arm slightly above the maze. Mice were placed with their front paws in the center of the square maze facing a closed arm. Each mouse was filmed for 5 min and scored for time spent in the open arms, time spent in the closed arms, number of entries to open arms, and number of entries into closed arms^100–102^. Animals were considered to be in an arm when the first half of the body was within the arm^3, 103^. The center zone connecting all arms was excluded^6, 104^.

### Balance beam test (BBT)

The mice were required to traverse an elevated, narrow aluminium beam (1 cm diameter, 100 cm long and 50 cm above a foam pad to cushion falling mice) to reach an enclosed escape box. Mice were first trained (4–5 trials) and were tested (3 trials) on the next day. We calculated the mean latency (sec), distance travelled (cm), number of foot slips, number of turns, and number of falls across the 3 testing trials ^6, 105^.

### Morris water task (MWT)

The water task consisted of a pool (153 cm diameter) filled with water (23–25 ± 1 °C) up to a level of ~15 cm from the top edge of the tank. The water was made opaque by non-toxic white tempura paint. The pool was located in a room rich with distal cues, which remained unobstructed throughout the duration of the experiment^106^. During all hidden platform trials, the platform was submerged ~1.0 cm under the surface of the water. The tank was divided into four quadrants, 1, 2, 3 and 4 using software, with starting points at north, west, east and south. The starting positions of the mice were located at the intersection of the quadrants, 4–5 cm away from the edge of the tank. Animals were trained with 4 trials per day for 8 consecutive days under regular room light (Water2100 Software vs.7, 2008). Each trial began with the mouse being placed in the pool in a pseudo-random sequence at one of the four cardinal compass positions around the perimeter of the pool. The acquisition trial was started by placing a mouse facing the wall of the tank at one of the 4 starting locations. Testing was stopped after the mouse reached the platform or, if the mouse did not find the platform, at the 60 second trial time limit. If a mouse found the platform within this 60 sec period, it was allowed to remain on the platform for five additional seconds. If it did not find the platform during the selected time, it was placed onto the platform for 15 sec by the experimenter before being returned to his home cage. Data were recorded using an automated tracking system (HVS Image Hampton, U.K.). Following each swim trial, the animal was dried with a soft fabric cloth and placed back into the home cage where it was allowed to rest for at least 5 min before the start of the next swim trial. The swim time (sec), swim speed (m/s), and swim distance (m) were calculated for analysis^31, 79^.

The probe trial was carried out on the ninth day, in which the platform was removed and each mouse was allowed to swim freely for 60 sec. In order to preclude the possible impact of working memory on retention ^107^ this trial was performed 24 hours after the last acquisition trial. The time spent in the quadrant where the platform had been located was measured.

### Statistical analysis

All statistical analyses were performed using SPSS Statistics 24.0 at a significance level of 0.05 or better. Normally distributed data were analyzed using the Kolmogorov–Smirnov test. Multivariate analysis of variance (MANOVA) was conducted to compare the three groups in terms of different parameters of the behavioral tests. A two-way MANOVA was performed to compare the three groups in CORT levels one day before starting and one day after finishing all behavioral tests, and the delta CORT level (difference between the two CORT levels measured) as well. To determine the possible effect of swim speed on the swim time, a multivariate analysis of covariance (MANCOVA) was done to compare the three groups in swim time where swim speed was considered as a covariate. A repeated measures ANOVA was conducted to compare the eight-days of training in the MWT, as well as the CORT levels one day before starting and one day after finishing all behavioral tests in every group. The F values, p values, estimations of the effect size (partial η^2^), and observed power have been reported for the statistical analyses. For multiple comparisons of group means in each measurement, the Tukey *post-hoc* test was performed.
